# Quantification of myocardial viability in diffuse and contiguous infarction using 3D inversion recovery MRI: Validation against microscopy

**DOI:** 10.1186/1532-429X-16-S1-P80

**Published:** 2014-01-16

**Authors:** Robert Jablonowski, Mark W Wilson, Noor Joudi, Maythem Saeed

**Affiliations:** 1 Department of Radiology and Biomedical Imaging, School of Medicine, University of California San Francisco, San Francisco, California, USA

## Background

Sheifer et al indicated that undetected infarction account for at least 20% of all infarctions and carry a prognosis as poor as detected ones [1]. Large myocardial infarction has been measured using both 3D and 2D delayed enhancement MRI, which was compared with histochemical staining [2]. The purpose of this MRI study was to 1) measure diffuse and large infarction size using delayed contrast enhanced 3D inversion recovery (IR) gradient echo (GRE) in beating and non-beating swine hearts and 2) compare the 3D measurements against 2D-IR GRE, histochemical staining and microscopic histopathology.

## Methods

Pigs were subjected to: 1) diffuse infarction by infusing 32 mm^3 ^microemboli in the LAD (group I, n = 7), 2) large infarction by occluding LAD for 90 min (group II, n = 7) or 3) combined large and diffuse infarction by occluding LAD for 90 min and infusion of 32 mm^3 ^microemboli (group III, n = 7). Three days following coronary intervention, contrast enhanced 3D (TE/TR/TI = 2.3/4.8/230-240 ms) and 2D (TE/TR/TI = 1.8/6.8/230-240 ms) was performed for measuring myocardial infarction in beating hearts. The animals were then euthanized inside the scanner and reimaged using the same imaging sequences. At postmortem, histochemical triphenyltetrazolium chloride (TTC) and microscopic histopathology (hematoxylin-eosin) stains were used for validation.

## Results

**Figure 1:**
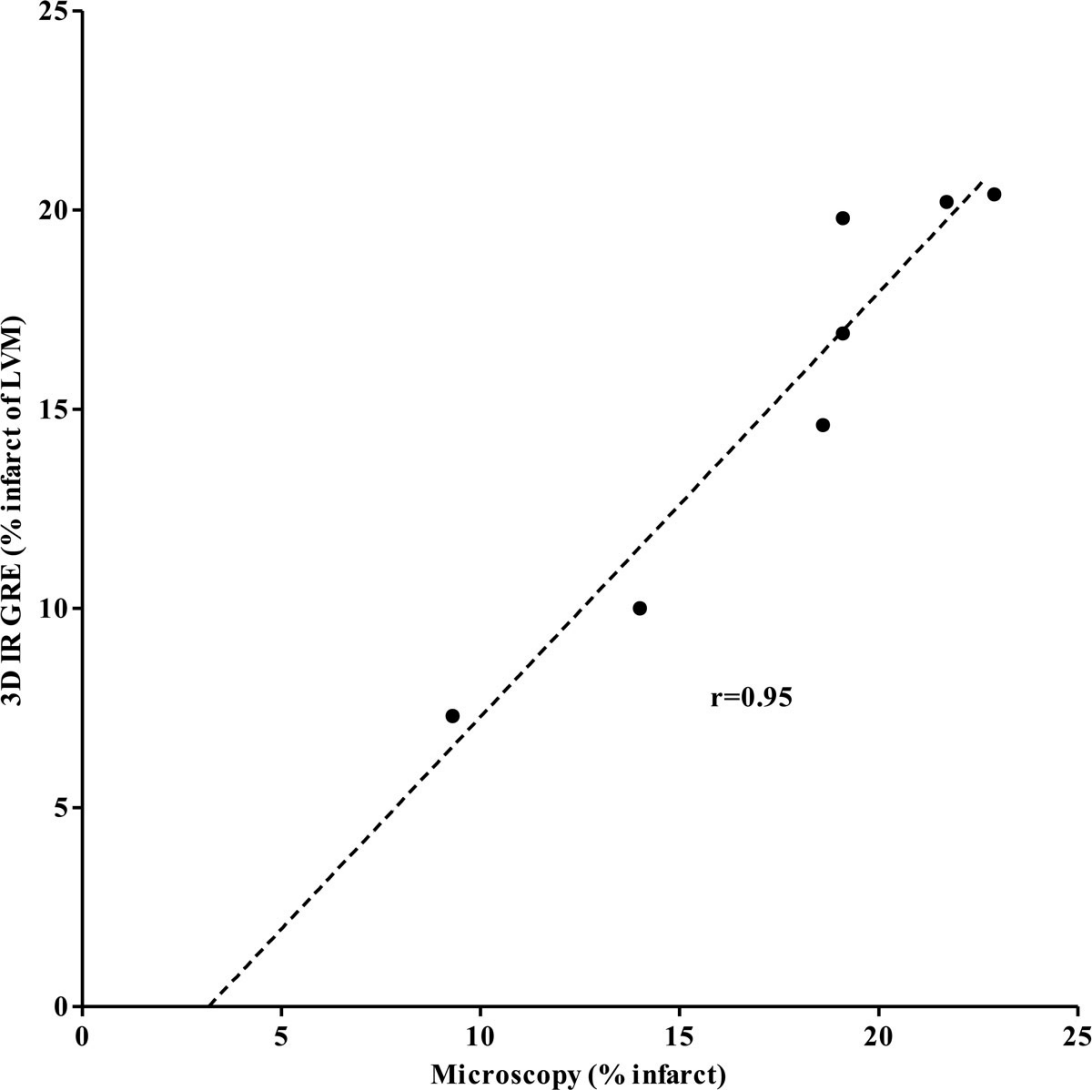
**Correlation between myocardial infarction measured on 3D MRI in beating hearts and microscopy (y = 1.1 × -3.5)**.

There was no significant difference in infarction size between delayed contrast enhanced 3D and 2D MRI in both beating and non-beating hearts (Table 1). Furthermore, theses sequences demonstrated the gradients in infarction size as a function of insult severity. In beating hearts close correlations and agreements were found between infarction size measured on 3D and 2D MRI (r = 0.81-0.95 for all groups, bias: group I = 0.033 ± 1.2%, group II = 0.13 ± 1.2%, group III = -0.17 ± 1.8%). Acute large infarction was overestimated on 3D and 2D MRI in beating hearts compared with microscopy due to the inclusion of edematous border zone. Figure 1 demonstrates a close correlation between 3D MRI in beating hearts and microscopy of combined large and diffuse infarction (group III), but 3D MRI underestimated true myocardial infarction size (bias: -2.2 ± 1.6%) due to the inherent limited spatial resolution and the small islands of necrosis in the area at risk.

**Table 1:** Comparison between 2D, 3D-LGE MRI, histochemical staining and histopathological infarct size.

	3D-IR GRE	2D-IR GRE	TTC	Microscopy
**Beating heart**				

Group I	9.0 ± 0.6	9.0 ± 0.3		

Group II	14.4 ± 0.6*^+^	14.3 ± 0.5*^+^		

Group III	16.0 ± 1.8*	15.8 ± 1.6*		

**Non-beating heart**				

Group I	9.0 ± 0.4	9.2 ± 0.7	8.4 ± 0.4	

Group II	14.6 ± 0.6*	14.1 ± 0.5*	14.9 ± 1.8*	13.3 ± 0.5

Group III	16.1 ± 1.9*	16.1 ± 2.1*	16.0 ± 1.6*	17.8 ± 1.8^#$^

*p < 0.05 compared with group I, ^#^p < 0.05 compared with coherent 3D and 2D, ^;$^p < 0.05 compared with group II, ^;+^p < 0.05 compared with microscopy of the same cohort.

## Conclusions

Myocardial infarction measured on 3D MRI is highly correlated and in a good agreement with infarction measured microscopically. This imaging sequence has the potential to measure diffuse and large acute myocardial infarction and has minimal motion artefacts.

## Funding

N/A
